# PSSweb: protein structural statistics web server

**DOI:** 10.1093/nar/gkw332

**Published:** 2016-05-12

**Authors:** Thomas Gaillard, Roland H. Stote, Annick Dejaegere

**Affiliations:** 1 Laboratoire de Biochimie, École Polytechnique, CNRS, Université Paris-Saclay, 91128 Palaiseau cedex, France; 2 Department of Integrative Structural Biology, Institut de Génétique et de Biologie Moléculaire et Cellulaire, Institut National de la Santé et de la Recherche Médicale U964, Centre National de la Recherche Scientifique UMR 7104, Université de Strasbourg, 67404 Illkirch, France

## Abstract

With the increasing number of protein structures available, there is a need for tools capable of automating the comparison of ensembles of structures, a common requirement in structural biology and bioinformatics. PSSweb is a web server for protein structural statistics. It takes as input an ensemble of PDB files of protein structures, performs a multiple sequence alignment and computes structural statistics for each position of the alignment. Different optional functionalities are proposed: structure superposition, Cartesian coordinate statistics, dihedral angle calculation and statistics, and a cluster analysis based on dihedral angles. An interactive report is generated, containing a summary of the results, tables, figures and 3D visualization of superposed structures. The server is available at http://pssweb.org.

## INTRODUCTION

As the number of available protein structures continues to increase (1), it is becoming more critical to have tools that efficiently allow the comparison and analysis of structural ensembles. Such analysis can be useful for understanding protein flexibility, to select templates for homology modeling, or to study the effects of ligand binding or mutations. When comparing structures with similar but different sequences, it is useful to establish a relationship between the residues of the structures. One way to obtain such correspondence is to perform a multiple alignment of the sequences. The sequence alignment is useful in itself to highlight conserved regions and derive structural, evolutionary or functional hypotheses. The comparison of the structures can then be addressed, first, through structure superposition, which provides additional insights than sequence alignment alone. Once superposed, quantitative information on the structural variability can be obtained by calculating the average and standard deviation of atomic Cartesian coordinates. Complementary information can be obtained from internal coordinates, such as the dihedral angles of the backbone (ϕ, ψ, ω) and the sidechains (χs). Finally, a cluster analysis can be performed to classify the structures.

We present here PSSweb (http://pssweb.org), a web server for protein structural statistics. Given a set of protein structures, PSSweb automatically performs multiple sequence alignment, structure superposition, calculates Cartesian and dihedral coordinate statistics and performs cluster analysis. Results are presented in an HTML report containing a convenient summary with interactive figures and tables, as well as 3D visualization of superposed structures. PSSweb is based on PSS (2), a previously published command-line program, and is intended for facilitating its use by a wider audience. In the original PSS article, we illustrated the interest of the program through several examples of application to pertinent biological problems. A search of other programs able to perform the same analysis showed that, to date, there are none that can carry out equivalent tasks (see the PSS article (2) for more details).

In the next sections, the improvements to the PSS program are first presented, followed by a description of the PSSweb server and by an example of application. A detailed description of the inner functioning of PSS, the stand-alone program behind PSSweb, is available from the PSS web page (http://thomasgaillard.fr/pss) and paper (2).

## IMPROVEMENTS TO THE PSS PROGRAM

The development of PSSweb prompted improvements to the PSS program that are summarized here. We have included more alternatives for both multiple sequence alignment and structure superposition. Multiple sequence alignment can now be performed with the Clustal Omega program (3), and structure superposition with the Theseus program (4). Superposed structures can now be interactively visualized directly in the HTML report thanks to JSmol (5). Finally, the format of the chain field in the Structure list has been enriched with first chain and multiple chains autodetection.

## DESCRIPTION OF THE WEB SERVER

**Figure 1.**
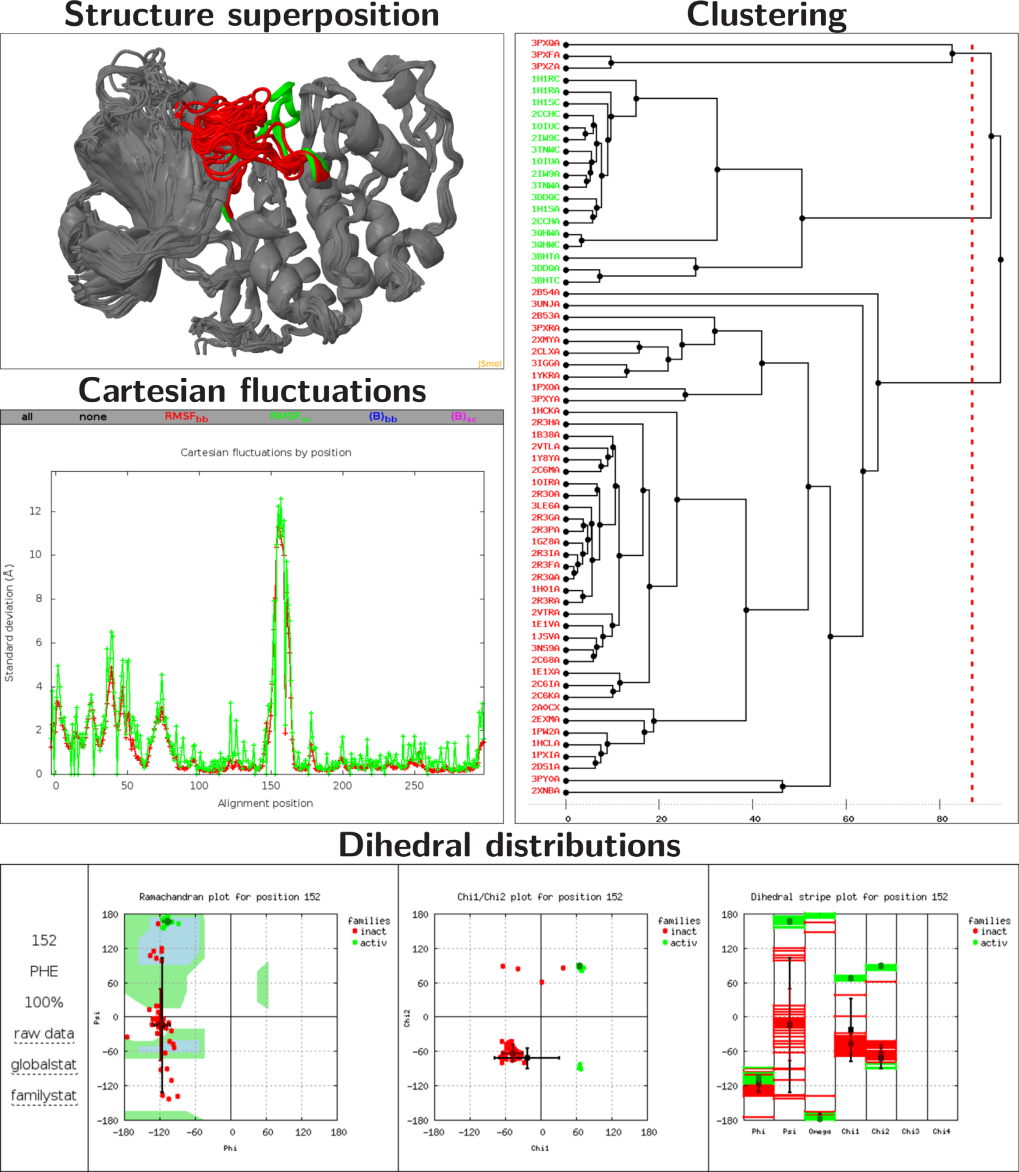
Overview of PSSweb capabilities on an example application. **Structure superposition**. Superposition of 64 CDK2 structures on 1HCK:A, visualized with a JSmol (5) viewer. The L12 helix and T-loop region (residues 144–166), which undergoes a major conformational change between inactive and active states, is highlighted in red and green for the inactive and active states, respectively. **Cartesian fluctuations**. Standard deviation of Cartesian coordinates, for backbone or sidechain atoms as a function of the alignment position. Zero points in sidechain fluctuations correspond to Gly residues. Dihedral coordinate fluctuations can also be obtained. **Dihedral distributions**. Dihedral distributions for the residue Phe152 of inactive (red) and active (green) structures. Average values and standard deviations, by family and globally, are also indicated. Left: Ramachandran plot. Center: χ_1_/χ_2_ plot. Right: dihedral stripe plot. **Clustering** The tree is obtained from hierarchical clustering with the maximum-linkage method. Variables taken into account are the ϕ and ψ angles of the L12 helix and T-loop region. Inactive and active structure PDB codes are respectively written in red and green.

PSSweb takes as input an ensemble of PDB files of protein structures. It performs a multiple sequence alignment, and computes structural statistics for each position of the alignment. Different optional functionalities are proposed: structure superposition, Cartesian coordinate statistics, dihedral angle calculation and statistics, and a cluster analysis based on dihedral angles. An interactive report containing a summary of the results with figures and tables is generated. An overview of PSSweb capabilities illustrated by an example of application is available in Figure 1. The web server includes a detailed help page and commented examples explaining how to interpret the results. A testcase can be easily loaded with a ‘Load example’ button. A link to the result page is provided at the time of job submission, allowing the user to bookmark it and access the results later. A link to a ZIP file of the entire result directory is also provided for download. The server code is compliant with HTML and CSS standards.

### Input

Input data for PSSweb consists of a set of PDB files containing protein structures and of an optional list of positions.

#### Structure list

A list of structures is needed as input. Each entry consists of a PDB file basename, an optional chain identifier and an optional user-defined family name. Autodetection of the first chain and of all chains of a PDB file is possible with ‘first’ (default) and ‘*’, respectively. A blank chain identifier is entered as ‘_’. If the basename has the format of a PDB code, an attempt is made to download the file from the PDB server. It is also possible to upload custom PDB files or ZIP of PDB files. Only standard PDB ATOM records are treated by PSSweb. When an atom has alternate locations, only the first one is considered. Only the first model of nuclear magnetic resonance (NMR) PDB files is read.

#### Position list

It is sometimes of interest to focus the analysis on a specific subset of amino acid positions. To provide for this option, a list of positions can be entered. The Position list restricts the list of amino acid positions and structural variables of interest for certain analyses. Each entry consists of number ranges determining which positions are included in the cluster analysis and in dihedral distribution plots, and of dihedral angles to be included in clustering. The reference for position numbering is the multiple sequence alignment.

### Multiple sequence alignment

Obtaining a multiple alignment of input protein sequences is an essential first step in the PSSweb workflow. PSSweb is indeed meant to analyze ensembles of proteins with similar, but different sequences and it is necessary for subsequent analyses to establish a correspondence between amino acid positions of the different sequences. We note that some structural alignment methods do not require a sequence alignment as input. We propose four different approaches to multiple sequence alignment: PDB numbering, sequence alignment with Clustal Omega, sequence and sequence/structure alignment with MODELLER. The simplest method consists of deriving the multiple sequence alignment from PDB numbering. It is assumed in this case that residues are numbered in a consistent way. The advantage is speed and no risk of error in the alignment, in particular at gap borders. If the numbering is not coherent, this method will give unreliable results and so proper checking should be performed when using PDB numbering. The Clustal Omega program (3) is a fast and scalable general multiple sequence alignment program, default options are employed. The sequence and sequence/structure alignment methods with MODELLER rely on the SALIGN (6–8) module of the MODELLER program (9). Details on the parameters used with SALIGN are available in the PSS documentation. Sequence alignment methods are the recommended default choice. The sequence/structure method is recommended for more distantly related sequences. It is not possible to define a general criterion to decide which sequence alignment method will work best, experimentation is encouraged and generated alignments should be assessed for quality. A fifth possibility offered is to upload a custom multiple sequence alignment. The alignment positions are numbered starting from one by default. This numbering is used to select positions in subsequent analyses, through the Position list. It can be shifted with the ‘First alignment position’ option.

### Superposition of structures

Superposition of structures can be performed with the Theseus program (4) or with the MODELLER program (9). Theseus uses a maximum likelihood approach, while MODELLER uses an iterative least-square minimization algorithm. The reference structure for the superposition can be defined, otherwise the first structure is taken. Interactive 3D visualization of the superposed structures is possible thanks to a JSmol (5) viewer included in the results page. The rendering style and coloring mode can be modified and the individual structures can be displayed or hidden. Visualization with JSmol can be slow for large ensembles or large structures. It is also possible to download the result directory thanks to the ZIP file provided and to visualize the superposed structures offline with the PyMOL (10) and VMD (11) scripts included.

### Cartesian analysis

Structural statistics can be computed from Cartesian coordinates. It is assumed that structures have been previously superposed. Figures of root mean square fluctuations of Cartesian coordinates and average B-factors, for backbone or sidechain atoms, as a function of the alignment position are prepared. These figures are superposed by default, to ease comparison, each plot can be hidden/displayed. In addition, a sortable and scrollable table containing the data is provided. The Cartesian analysis provides a characterization of structural variations in the protein set along the sequence, allowing the identification of the most flexible and rigid regions. Note that the B-factor field may be used to store other quantities, in particular in NMR structures.

### Dihedral analysis

Structural statistics can also be computed from the dihedral angles. Figures and tables of ϕ, ψ, ω, χ_1_, χ_2_, χ_3_ and χ_4_ dihedral angle standard deviations as a function of the alignment position are prepared. Additionally, Ramachandran (ϕ/ψ), χ_1_/χ_2_ and individual dihedral angle distribution plots are prepared for each alignment position defined in the Position list. If different families were defined in the Structure list, different colors are used to distinguish points belonging to each family. Displaying the distribution plots of many positions can produce a substantial HTML page. Note that the backbone dihedral angle ϕ is not defined at the N-terminal side of contiguous residues, while ψ and ω are not defined at the C-terminal side. The dihedral coordinates have an internal nature and are thus not affected by structure superposition. The dihedral analysis provides similar but complementary information to the Cartesian analysis (see the PSS article (2) for a discussion on this).

### Clustering

The structures can be subjected to a hierarchical clustering analysis, based on the Algorithm::Cluster Perl module, an interface to the C Clustering Library (12). Structural variables taken into account are dihedral angles and can be controlled with the Position list. The circular distance between selected dihedral angles is the metric. Maximum linkage is used. A tree is calculated and the clusters are obtained by cutting the tree according to the chosen cluster radius option (default is 60°). The radius parameter corresponds to the minimum distance allowed between clusters. A dendrogram figure is prepared to visualize the clustering tree obtained, with the chosen cutting radius as a dotted line. To facilitate the setup of another PSSweb run with a definition of families corresponding to the identified clusters, a Structure list file with cluster numbers in the family column is provided.

## EXAMPLE OF APPLICATION

As an example of application, PSSweb was used to analyze structural variations in an ensemble of 64 protein structures of the human cyclin-dependent kinase 2 (CDK2) (13–15) (see Figure 1). CDK2 is an interesting system to illustrate structural variations of a protein because of the existence of two well-defined inactive and active states and the large amount of structural information available. PSSweb was able to identify the most structurally variable regions of CDK2. A cluster analysis focusing on these elements was able to map the structures into clusters that exactly match the inactive and active states of the protein. The report corresponding to this example is available at this URL: http://pssweb.org/examples/cdk2. This CDK2 example and two others involving nuclear receptors are discussed with more details in the PSS article (2) and in the PSSweb server ‘examples’ page.

## CONCLUSION

The PSSweb server (http://pssweb.org) for protein structural statistics was presented. PSSweb takes as input an ensemble of PDB files of protein structures, performs a multiple sequence alignment and proposes different optional functionalities: structure superposition, structural statistics in both Cartesian and dihedral coordinates and cluster analysis. The results are presented in an interactive HTML page, containing figures, tables, 3D visualization of superposed structures and access to raw data. PSSweb is based on the already published PSS program (2). The web server interface greatly facilitates access to PSS for a wider audience by avoiding the installation step and the command-line usage requirement. Further improvements will be guided by user demands. The automated comparisons of protein structures performed by PSSweb are of the greatest interest in structural biology, homology modeling, ligand and protein design. The relevance of these analyses will increase with the growing number of protein structures available.
